# Initiation of hnRNPA1 Low-Complexity Domain Condensation Monitored by Dynamic Light Scattering

**DOI:** 10.3390/ijms25136825

**Published:** 2024-06-21

**Authors:** Phoebe S. Tsoi, Josephine C. Ferreon, Allan Chris M. Ferreon

**Affiliations:** Department of Biochemistry and Molecular Pharmacology, Baylor College of Medicine, Houston, TX 77030, USA

**Keywords:** hnRNPA1, biomolecular condensates, nano-condensates, nano-clusters, protein phase separation, neurodegenerative diseases

## Abstract

Biomolecular condensates (BMCs) exhibit physiological and pathological relevance in biological systems. Both liquid and solid condensates play significant roles in the spatiotemporal regulation and organization of macromolecules and their biological activities. Some pathological solid condensates, such as Lewy Bodies and other fibrillar aggregates, have been hypothesized to originate from liquid condensates. With the prevalence of BMCs having functional and dysfunctional roles, it is imperative to understand the mechanism of biomolecular condensate formation and initiation. Using the low-complexity domain (LCD) of heterogenous ribonuclear protein A1 (hnRNPA1) as our model, we monitored initial assembly events using dynamic light scattering (DLS) while modulating pH and salt conditions to perturb macromolecule and condensate properties. We observed the formation of nanometer-sized BMCs (nano-condensates) distinct from protein monomers and micron-sized condensates. We also observed that conditions that solubilize micron-sized protein condensates do not solubilize nano-condensates, indicating that the balance of forces that stabilize nano-condensates and micron-sized condensates are distinct. These findings provide insight into the forces that drive protein phase separation and potential nucleation structures of macromolecular condensation.

## 1. Introduction

Protein condensation is a process by which proteins partition into a protein-rich phase, distinct from the more dilute bulk phase. The field of protein condensation has gained extraordinary popularity within the recent decade, with particular interest in biomolecular condensates (BMCs): cellular protein condensates with functional roles in spatiotemporal organization and regulation of biological processes [1,2,3], as well as involvement in diseases, such as neurodegeneration and cancer [2,4,5,6].

Liquid condensates have been the focal point of functional BMC research, with well-established cellular liquid condensates categorized as ‘membraneless organelles’ (MLOs). Structures such as stress granules, p-bodies, Cajal bodies, and paranuclear speckles form naturally in the cell and sequester proteins, RNAs, and other cellular components into dense liquid phases to regulate biological processes and/or increase cell viability during stress [1,2,3]. Although functional BMC research focuses primarily on liquid condensates, solid condensates have also been shown to have biological functions. The term ‘amyloid’ is strongly associated with neurodegeneration. However, an amyloid is a structure, defined by cross-β-sheets formed by multitudes of the same protein whose substructure consists of beta-sheets running perpendicular to the fiber axis [7]. For example, some amyloids are involved in the sorting and storage of peptide hormones in the Golgi, and the inactivation of toxic proteins, such as the major basic protein, found in eosinophils and involved in immune activity [7,8].

Pathologic condensates, such as Lewy Bodies and neurofibrillary tangles, are irreversible protein aggregates observed in neurodegenerative disease [5]. There is evidence that these solid pathologic condensates originate from liquid condensates. Under stressful or pathological conditions, liquid condensates can undergo a liquid-to-solid phase transition, resulting in protein fibrillation [4,9].

BMC formation is driven by liquid–liquid phase separation, a process that has been studied in vitro by methods such as optical microscopy and turbidity assays [10,11]. The formation of these micron-sized droplets was thought to be initiated by classical nucleation theory. Briefly, molecules in solution at a concentration above a calculatable saturated concentration will favor the formation of a new phase [12,13,14]. According to classical nucleation theory, the probability of molecular assemblies at subsaturated concentrations is negligibly small [15]. However, nanometer-sized condensates have been observed in subsaturated concentrations for a myriad of proteins, including lysozyme [16,17], Tau [18], and FUS [15]. Apart from size, there has been minimal research investigating the distinction between nanometer-sized and micrometer-sized condensates.

To distinguish micron- and nanometer-sized condensates, here, these condensates will now be referred to as micro-condensates and nano-condensates, respectively. Nano-condensates are not amenable to detection by optical microscopy and turbidity assays used to study micro-condensates. Thus, we have used dynamic light scattering (DLS), a technique highly sensitive to size distributions of nanometer-scale molecular populations in solution, as our principal technique to study nano-condensates. Here, we monitor the formation of nano-condensates for 5 and 20 μM hnRNPA1^LCD^ in pH conditions ranging from pH 4 to 8 and in salt concentrations ranging from 0 to 500 mM (NaCl). We identified conditions where the protein either exists in monomeric state, forms nano-condensates, or coexists as nano-condensates and micro-condensates. Nano-condensate size positively correlates with increases in salt concentration and protein concentration, with significantly larger changes in nano-condensate size correlating with the presence of micro-condensates. We also observed that conditions that dissolve micro-condensates reduce nano-condensate size, but do not fully dissolve nano-condensates. These findings suggest that electrostatic interactions drive hnRNPA1^LCD^ nano-condensate formation, balanced by solvent/hydrophobic interactions, with nano-condensates being early intermediate, potentially nucleation, structures in the macromolecular phase separation process.

## 2. Results

### 2.1. hnRNPA1^LCD^ Monomers, Nano-Condensates, and Micro-Condensates Detected via DLS

**Figure 1:**
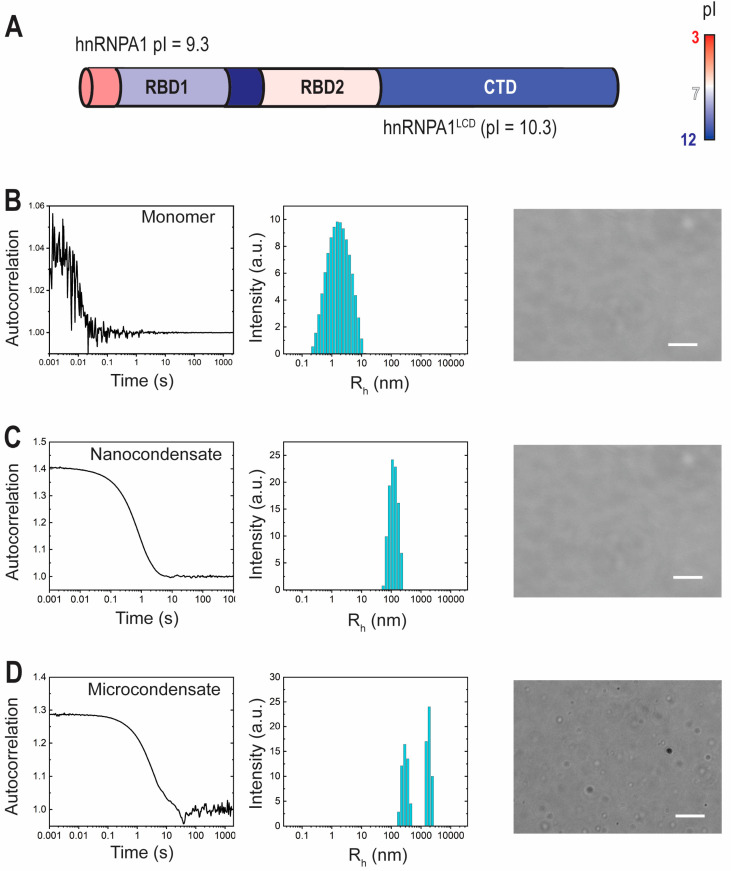
hnRNPA1^LCD^ monomer, nano-condensate, and micro-condensate populations. (**A**) hnRNPA1 domain organization. hnRNPA1 consists of two RNA-binding domains (RBDs) and a C-terminal prion-like low-complexity domain (LCD). Adapted from Tsoi et al. [19]. Representative conditions where major hnRNPA1^LCD^ populations are primarily monomeric (**B**), 20 μM hnRNPA1^LCD^, water), primarily nano-condensates (**C**), 20 μM hnRNPA1^LCD^, 0 mM NaCl, pH 7, αβγ buffer), or a coexistence of nano-condensates and micro-condensates (**D**), and 20 μM hnRNPA1^LCD^, 200 mM NaCl, pH 7, αβγ buffer) was observed using DLS and optical microscopy. Scale bar = 10 μm.

DLS and optical microscopy were used to observe the formation of nano-condensates and micro-condensates of the low-complexity domain of the heterogeneous nuclear ribonucleoprotein hnRNPA1 (hnRNPA1^LCD^). hnRNPA1 is an RNA-binding protein that consists of two structured N-terminal RNA-binding domains (RBD1 and RBD2) and an intrinsically disordered C-terminus (Figure 1A). We have previously characterized the electrostatic modulation of hnRNPA1 micro-condensate formation by varying pH conditions, as well as protein, RNA, and salt concentrations [19]. Here, we identified conditions where hnRNPA1^LCD^ exists as a monomer, forms nano-condensates, or forms both nano-condensates and micro-condensates.

Populations of hnRNPA1^LCD^ can clearly be experimentally distinguished as monomeric protein, nano-condensates, or combinations of nano-condensates and micro-condensates (Figure 1B–D). Protein monomers were observed at 20 μM hnRNPA1^LCD^ in water, characterized by a fast autocorrelation decay, corresponding to a hydrodynamic radius of 2.4 nm, and an absence of visible protein condensates via microscopy (Figure 1B). Protein nano-condensates were observed at 20 μM hnRNPA1^LCD^ in 10 mM each of sodium acetate, sodium phosphate, and glycine (αβγ buffer), and 0 mM of NaCl, pH 7 (Figure 1C), exhibiting a slower autocorrelation decay corresponding to a hydrodynamic radius of 135 nm. In conditions where micro-condensates were visible via microscopy (20 μM hnRNPA1^LCD^ in 200 mM NaCl, pH 7, αβγ buffer; Figure 1C), nano-condensates were also detectable via DLS. A slow autocorrelation decay was observed, with an irregular tailing end that did not reach a flat baseline during the experimental timescale. Using the regularization method [20], two hydrodynamic radii were extrapolated from DLS: one at approximately 350 nm, and the other at >1000 nm. These two peaks suggest that nano-condensates and micro-condensates form and coexist in some conditions.

### 2.2. Electrostatics Modulate Nano-Condensate Formation

**Figure 2:**
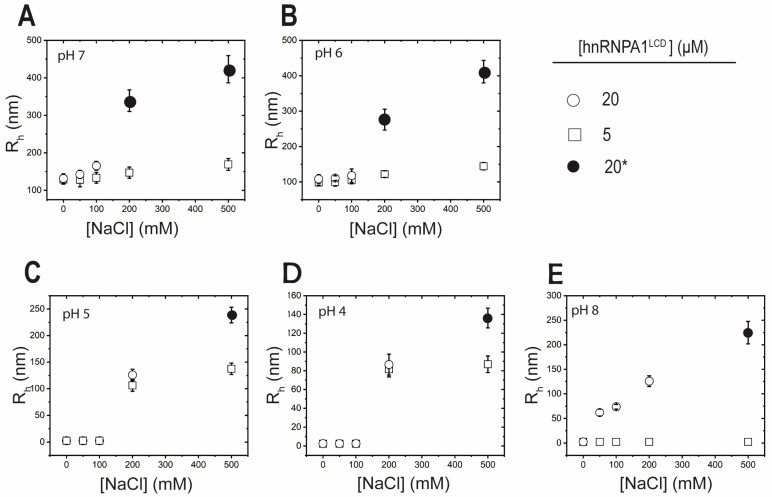
Micro-condensate and nano-condensate electrostatic modulation. (**A**) hnRNPA1^LCD^ monomer, nano-condensate, and micro-condensates observed for 20 μM and 5 μM protein in 0–500 μM NaCl, αβγ buffer, pH 7, (**B**) pH 6, (**C**) pH 5, (**D**) pH 4, and (**E**) pH 8. Circles refer to 20 μM hnRNPA1^LCD^ and squares refer to 5 μM hnRNPA1^LCD^. * Filled circles refer to the presence of micro-condensates.

By varying protein and salt concentrations, along with pH conditions, we were able to observe several phase separation trends. Nano-condensates formed in 10 mM neutral pH buffer without salt (Figure 2A), with a hydrodynamic radius of ~135 nm for 20 μM and ~128 nm for 5 μM hnRNPA1^LCD^. As the salt concentration increased, the hydrodynamic radius increased from ~135 to 420 nm for 20 μM hnRNPA1^LCD^ and from ~128 to 170 nm for 5 μM hnRNPA1^LCD^. At higher salt concentrations (200–500 mM NaCl), 20 μM hnRNPA1^LCD^ formed micro-condensates, but 5 μM hnRNPA1^LCD^ did not. Similarly, at pH 6 (Figure 2B), the same trends were observed for 20 and 5 μM hnRNPA1^LCD^. In 0 mM salt conditions, nano-condensates with a hydrodynamic radius of ~108 nm formed for 20 μM and ~98 nm for 5 μM hnRNPA1^LCD^. As salt increased, the hydrodynamic radius increased from ~108 to 410 nm for 20 μM hnRNPA1^LCD^ and ~98 to 144 nm for 5 μM hnRNPA1^LCD^. Again, we observed that at higher salt concentrations (200–500 mM), 20 μM hnRNPA1^LCD^ formed micro-condensates but 5 μM hnRNPA1^LCD^ did not. Interestingly, at pH 5 (Figure 2C), nano-condensates did not form at salt concentrations < 200 mM for both 20 and 5 μM hnRNPA1^LCD^. From 200 mM to 500 mM NaCl, the hydrodynamic radii of nano-condensates increased from ~125 to 238 nm for 20 μM hnRNPA1^LCD^ and from ~106 to 137 nm for 5 μM hnRNPA1^LCD^. Similarly, at pH 4 (Figure 2D), nano-condensates did not form at salt concentrations < 200 mM for both 20 and 5 μM of protein. For pH 4 and 5 conditions, 20 μM hnRNPA1^LCD^ formed micro-condensates only at a 500 mM salt concentration. From 200 mM to 500 mM NaCl, the hydrodynamic radii of nano-condensates increased from ~86 to 136 nm for 20 μM and ~81 to 87 nm for 5 μM hnRNPA1^LCD^. Lastly, at pH 8 conditions (Figure 2E), nano-condensates did not form in the presence of salt for 5 μM hnRNPA1^LCD^. At 50 mM NaCl, 20 μM hnRNPA1^LCD^ formed nano-condensates with a hydrodynamic radius of ~60 nm. As the salt concentration increased, the hydrodynamic radius increased from ~60 to 225 nm. At 500 mM NaCl, 20 μM hnRNPA1^LCD^ formed micro-condensates.

### 2.3. 1,6-Hexanediol Completely Solubilizes Micro-Condensates, but Not Nano-Condensates

**Figure 3:**
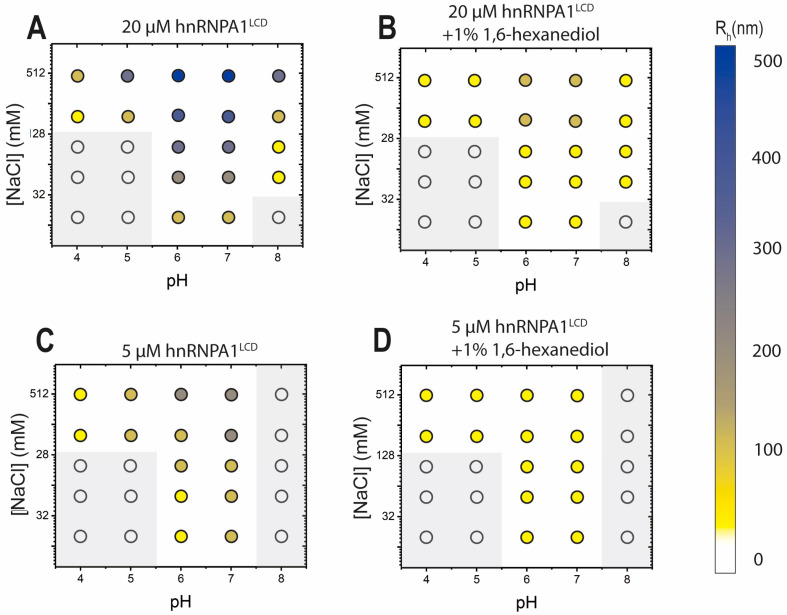
Hexanediol solubilized micro-condensates and decreased nano-condensate size. (**A**) hnRNPA1^LCD^ monomer and nano-condensate formation for 20 μM protein in 0–500 mM NaCl, pH 4–8, αβγ buffer. (**B**) hnRNPA1^LCD^ monomer and nano-condensate formation observed for 20 μM protein in 0–500 mM NaCl, pH 4–8, αβγ buffer, in the presence of 1% 1,6-hexanediol. (**C**) hnRNPA1^LCD^ monomer and nano-condensate formation observed for 5 μM protein in 0–500 mM NaCl, pH 4–8, αβγ buffer. (**D**) hnRNPA1^LCD^ monomer and nano-condensate formation observed for 5 μM protein in 0–500 mM NaCl, pH 4–8, αβγ buffer, in the presence of 1% 1,6-hexanediol. Grayed-over open circles refer to conditions wherein only monomers were detected.

1,6-hexanediol is usually used to verify the reversible nature of micro-condensate formation [21,22]. Here, 1% 1,6-hexanediol (*v*/*v*) visibly dissolved micro-condensates, observable using optical microscopy and turbidity assays. We used DLS to observe the effect of 1,6-hexanediol on nano-condensates. In 0–500 mM NaCl, pH 4–8, αβγ buffer, 5 and 20 μM hnRNPA1^LCD^, 1% 1,6-hexanediol was added and mixed before immediate DLS measurement. After hexanediol addition, we observed a general decrease in the hydrodynamic radius (Figure 3A,C, compared to Figure 3B,D, respectively), culminating in sizes that correspond to nano-condensates. In the presence of 1,6-hexanediol, all hydrodynamic radii decreased to ~50–80 nm in size, except for 20 μM protein and 200–500 mM salt concentrations at neutral and pH 6 conditions, where the hydrodynamic radii decreased to ~100 nm in size. 

## 3. Discussion

Nano-condensates were observed as early as 1999, when Georgalis et al. detected three lysozyme populations in solution: monomers, a nucleating population, and a third group of fractal clusters [16]. Shortly thereafter, similar nano-condensates were also observed for lumazine synthase [23], glucose isomerase [24], insulin [25], and ferritin [26]. The first groups investigating nano-condensation were crystallographers, and thus their focus was on the nucleation of protein crystals. The crystallographers studying nano-condensation made the observation that nano-condensates appeared to nucleate crystals through a non-classical pathway [24,25,26]. In 2021, Martin et al. studied the kinetics of nano-condensation in supersaturated concentrations of hnRNPA1^LCD^ and found that small condensates (<100 nm) behaved distinctly from larger condensates (>100 nm) [27]. In 2022, Kar et al. made the observation that nano-condensation defies classic nucleation theory of micro-condensate formation [15]. Two-step nucleation theories were proposed, where an initial disordered precursor forms, which can then rearrange to form nuclei with different degrees of order [15,27,28,29].

**Figure 4:**
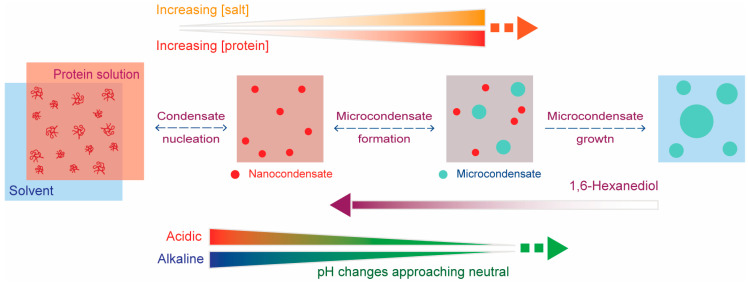
Model for hnRNPA1^LCD^ condensation nucleation. Observed nanometer-sized condensates (nano-condensates) were hypothesized as nuclei and initiation sites for biomolecular condensation. These nano-condensates exhibited slower, more linear growth characteristics as a function of monomer protein concentration, as compared to micron-sized condensates (micro-condensates). Phase separation was enhanced by increasing protein and salt concentrations, as well as pH changes approaching optimal protein charge (approximately neutral pH, for hnRNPA1^LCD^ in the solution conditions used). 1,6-hexanediol partially reversed the phase separation process, transforming micro-condensates to more stable nano-condensates.

Understanding the initiation of condensation is imperative to studying the mechanism of condensate formation and identifying where the switch from function to dysfunction can occur in the context of BMCs. Here, we identified conditions in which hnRNPA1^LCD^ favors either a monomeric state, forms nano-condensates, or coexists as combinations of both nano-condensates and micro-condensates, as summarized in Figure 4. Notably, the sizes of the nano-condensates appeared similar between 5 and 20 μM hnRNPA1^LCD^ until conditions where micro-condensates formed. This observation, where the protein concentration appeared to have a limited effect on nano-condensate size, has been observed in other protein systems [16,17,23,24,25,26]. The increased nano-condensate size in conditions where micro-condensates formed suggested that nano-condensates may be the nuclei for micro-condensation.

We also observed that increasingly acidic and basic conditions decreased nano-condensate size. Interestingly, 5 and 20 μM hnRNPA1^LCD^ concentrations shared similar salt thresholds for nano-condensate formation in acidic conditions, wherein 200 mM NaCl conditions allowed hnRNPA1^LCD^ to form nano-condensates at both pH 4 and 5. At pH 8 conditions, 5 μM hnRNPA1^LCD^ was unable to form nano-condensates even in high salt concentrations, but the 20 μM hnRNPA1^LCD^ condition was able to form nano-condensates with just 50 mM NaCl. These results are consistent with the stabilizing effect of charge screening on condensation. hnRNPA1^LCD^ is an alkaline protein with an isoelectric point at 10.3. In highly acidic conditions, charge screening mitigated the effect of charge repulsion between highly positively charged regions. At alkaline pH conditions, hnRNPA1^LCD^ approached neutral charge and exhibited reduced efficacy for charge screening to stabilize condensation, implying that condensate formation also requires some degree of electrostatic interactions. At pH 8, 5 μM hnRNPA1^LCD^ was unable to promote condensation, whereas 20 μM hnRNPA1^LCD^ required salt condensate stabilization.

Nano-condensates were not completely dissolved by 1,6-hexanediol, a solubilizing agent that interferes with the hydrophobic interactions that stabilize protein condensation [21,22] and solvophobic interactions in general [30]. However, nano-condensate size decreased overall in the presence of hexanediol. In general, this observation suggests that the balance of forces that stabilize nano-condensates is distinct from the balance of forces stabilizing micro-condensates. These observations also suggest that nano-condensates are primarily stabilized by electrostatic forces, whereas their micron-sized counterparts require additional solvophobic interactions to stabilize their larger size.

In summary, nano-condensates are distinct structures from micro-condensates and can be detected using DLS. Formation and initiation of nano-condensates and micro-condensates are governed by a delicate balance of electrostatic and, in general, solvophobic interactions. Some future directions of this study include further distinguishing nano-condensates and micro-condensates through differences in their physical/chemical properties. Distinguishing these condensates can also be accomplished by determining the growth mechanisms and the molecular organization of these structures. Another direction would be the aging and morphological evolution of nano-condensates and micro-condensates and observing the point at which liquid to solid transition can manifest. These findings would provide deeper insight into possible pathways to pathological aggregation, and potential avenues toward therapeutic intervention.

## 4. Materials and Methods

### 4.1. hnRNPA1^LCD^ Expression and Purification

Rosetta DE3 cells transformed with pET28a-H6t-A1c plasmids encoding human hnRNPA1^LCD^ (residues 186–320) were grown at 37 °C in an overnight subculture of Terrific Broth media containing kanamycin and chloramphenicol antibiotics. Saturated cells were added at a 1:200 dilution to 1 L TB media containing kanamycin. Cells were grown at 37 °C and induced with 1 mM IPTG when the culture reached an optical density between 0.8 and 1.0. The cells were grown overnight at 18 °C and harvested by centrifugation at 4000× *g* for 20 min. Cells were resuspended in lysis buffer (500 mM NaCl, 1 M guanidine hydrochloride (GdnHCl), and 50 mM sodium dibasic phosphate, mixed with 109 mM sodium phosphate buffer pH 8; final pH 7.8) and lysed using a homogenizer (Avestin, Ottowa, ON, Canada). Lysate was centrifuged at 4 °C for 2 h at 50,000× *g*, and the resulting supernatant was incubated with nickel agarose beads (GoldBio, St. Louis, MO, USA) for 30 min. The solution was then set up for gravity affinity chromatography. Lysate was allowed to pass through, and the beads were washed extensively in lysis buffer before protein was eluted in 2 M GdnHCl and 200 mM imidazole. Elution was diluted 2–3-fold with 0.2%TFA /2%ACN. An additional 2–2.5 mL of 10% TFA was added, and then the solution was spun down for 20 min at 6000× *g*. The supernatant was filtered with a 0.2-micron filter before HPLC. After HPLC purification, samples containing the target protein were lyophilized overnight. Lyophilized protein was dissolved in water to a final concentration of 0.5 mg/mL. Sodium acetate buffer was added to a 5 mM concentration, pH 5.2, and 5 mM Tris buffer, pH 8, was added to a final pH condition of ~6.5–7.0. TEV protease was added at a 1:20 molar ratio and incubated while rotating overnight at RT. The solution was then centrifuged at 6000× *g* for 20 min. The supernatant was diluted to a final condition of 10 mM imidazole and 1 M GdnHCl. The solution was applied to nickel agarose beads (GoldBio) for reverse-affinity chromatography. The flowthrough was collected and prepared for C18 HPLC. After HPLC, fractions containing the protein were collected, lyophilized, and stored at −30 °C before further use.

### 4.2. Dynamic Light Scattering

Dynamic light scattering (DLS) was conducted using the DynaPro Nanostar II (Wyatt Technology, Waters Corporation; Santa Barbara, CA, USA). DLS measures Brownian motion of the molecules in solution by analyzing intensity fluctuations of scattered light from an incident laser (658 nm). The fluctuations of scattered light were collected by orthogonal detectors and correlated with the diffusion coefficient (*D*), which is related to the hydrodynamic radius (*R_h_*) through the Stokes–Einstein equation:

$$R_{h}=\frac{kT}{6\pi \eta D}$$

where *k* is the Boltzmann constant, *T* is absolute temperature, and *η* is the viscosity of the solution.

Lyophilized hnRNPA1^LCD^ was dissolved in water before subsequent double filtration using 0.02 μm filters (Whatman, Cytivia, Maidstone, UK). Samples of varying protein (5–20 µM) and salt (0–500 mM) concentrations and pH conditions (pH 4–8) were prepared in αβγ buffer (10 mM each of sodium acetate, sodium phosphate, and glycine) and pipetted into a 45 µL quartz cuvette (Wyatt Technology, Waters Corporation; Santa Barbara, CA, USA). All solutions involved in sample preparation were double-filtered with 0.02 μm filters.

Unless otherwise specified, 10 DLS acquisitions were taken at 2 s intervals over 10 min at 25 °C, in triplicate. Condensate sizes were averaged and reported at each condition. Errors for reported values correspond to compounded standard deviation from the averaged acquisitions and technical triplicates.

### 4.3. Microscopy Imaging

Condensates were imaged using varying protein (5–20 µM) and salt (0–500 mM NaCl) concentrations and pH (4–8) conditions. 5 µL drops were pipetted onto 35 mm glass-bottom dishes (ibidi, Martinsried, Germany) and droplet formation was immediately monitored over one hour. Microscopy images were recorded at RT using an FL EVOS imaging system (Invitrogen, Waltham, MA, USA).

## Data Availability

The dataset is available from the authors upon request.
